# Polyester microfibers delay growth of cherry tomato (*Solanum lycopersicum var. cerasiforme*) throughout the lifecycle

**DOI:** 10.1371/journal.pone.0336191

**Published:** 2026-01-14

**Authors:** Natasha Djuric, Chelsea M. Rochman, Shelby H. Riskin

**Affiliations:** 1 Soil and Crop Sciences Section, School of Integrative Plant Science, Cornell University, Ithaca, New York, United States of America; 2 Department of Ecology and Evolutionary Biology, University of Toronto, Toronto, Ontario, Canada; Jiangsu University, CHINA

## Abstract

Agroecosystems are increasingly recognized as major basins for terrestrial microplastics. Many agricultural practices have led to high loading of plastic mulch films, synthetic microfibers, and other microplastics onto fields. There is demonstrated ability for microplastics to influence soil properties and plant productivity, but these effects are highly variable by species, soil, and life stage. Here, we conduct a lifecycle assessment of how polyester microfibers, a dominant biosolid contaminant, affect the development of cherry tomato (*Solanum lycopersicum var. cerasiforme*) in peaty growing medium. We distinguish the importance of physical and chemical characteristics of microfibers by comparing plants in soil containing microfibers at 0.5% soil weight, or soil watered with leachate isolated from microfibers. We find that polyester microfibers reduce emergence success by 11%, delay flowering and ripening time by several days, and lead to a reduction in biomass in adult plants. However, we observe no effect of chemical additives from microfibers on plant development. We also note a decrease in soil water holding capacity from microfibers. Overall, we conclude that physical microfiber properties or physical/chemical interactions are the likely drivers of biological effects. These findings emphasize that microfibers have impacts beyond mineral soils, and consequences at every stage of plant development.

## Introduction

Widespread dependence on plastic for commercial and personal use has made microplastics (plastic particles <5 mm in length) ubiquitous in the environment. While extensive research has focused on microplastic pollution in aquatic systems, microplastics in terrestrial systems long went understudied [1,2]. Yet, these systems are a critical global reservoir and pathway into aquatic environments. Moreover, microplastics on land have a suite of effects on soil and organisms [3,4].

Agroecosystems have garnered particular attention relevant to microplastics in terrestrial environments, as croplands are estimated to be a terrestrial microplastic reservoir ranking just below beaches, landfills, and urban spaces [5]. Terrestrial microplastics enter agricultural soils through farming practices like plastic mulching and sewage sludge application [4]. Different agricultural practices introduce different types of microplastics. For example, sewage sludge application is closely linked with the presence of synthetic microfibers such as polyester, nylon, and acrylic [6] in agricultural fields. These microfibers first enter wastewater through household laundering and as effluent from textile factories [7]. The wastewater treatment process retains up to 99% of microfibers in sewage sludge, which is subsequently applied onto farmland as a soil amendment. The remaining microfibers are discharged in water as treated effluent, which may be used for irrigation. Once applied, these microfibers have been shown to persist in agricultural soils for at least a decade [6,8].

Given the large quantities of sewage sludge applied to agricultural fields globally, ranging from 45 to 75% in some countries [9], it is critical to understand how microfibers affect terrestrial biology. The shape, flexibility, size, hydrophobicity, and charge of microfibers [10] may all affect soils and plants. Microfibers can influence soil bulk density and aggregate stability [11–13], soil moisture dynamics [10], and plant biomass accumulation [10,12]. However, these effects depends on soil type [14], plant species [15], and microfiber concentration [1,11], measured as percent weight per weight (w/w). For example, polyester microfibers at 0.5% w/w decreased soil bulk density in a clayey Vertisol but had no effect on a sandier Alfisol [14], and polyester microfibers at 0.4% w/w showed greater increases in water holding capacity than at 0.1% w/w [11]. Furthermore, microplastics may affect plant growth directly through adherence to the root surface, potentially reducing water and nutrient uptake or inducing toxicity [16]. Even under similar conditions, plants may respond differently to microfibers. In a community composition study, [17] found microfibers at 0.4% w/w to increase shoot mass of reedgrass (*Calamagrotis epigejos*) but decrease shoot mass of herb hawkweed (*Hieracium pilosella*). Chemicals can also dramatically affect plant responses. Chemical additives are added to plastic polymers to increase their lifespan and function. These compounds leach into the environment, as they are generally not tightly bound to the polymer [18]. Dyes, particularly azo dyes, readily release from fabric and have been shown to stunt plant growth and disrupt soil chemistry and microbes [18]. Additionally, benzotriazole and benzothiazole UV stabilizers, available in polyester textiles [19] may have plant-regulatory effects and be metabolized by plants into analogs of important growth hormones like auxin [20].

Overall, these studies demonstrate that microfibers can influence plant productivity through diverse effects on soil properties [10–14] water availability [10,11], direct physical contact [16], and plastic additives [18–20] and that these impacts vary by plant species [17]. The variable and sometimes contradictory directions of observed impacts underscores the need to continue examining new contexts and broadening our mechanistic understanding. Here, we attempt to distinguish physical and chemical effects on soil properties and plant growth by exposing the cherry tomato (*Solanum lycopersicum var. cerasiforme)* to polyester microfibers or only the chemical leachate from microfibers in peaty, organic soil. We follow a cohort of plants from germination to fruit ripening to better understand how effects vary across the lifecycle. We assess whether microfibers and/or their chemical leachates affect the emergence success, growth rate, reproductive phenology, and above and belowground biomass of cherry tomatoes. We also investigate whether soil property changes still account for any effects, as peaty conditions, which largely eliminate the water retention and root penetration constraints of many mineral soils, are an unexplored medium for soil structural changes from microfibers.

We used polyester (PET) because this microfiber type sheds the most when textiles are washed [21] and accounts for a large fraction of microplastics in wastewater treatment plant effluent [22], which is applied to agricultural soils. We grew cherry tomatoes since most recent studies examining microplastic effects on plant growth have focused on field crops such as wheat (*Triticum aestivum* L.), corn (*Zea mays* L.), and rice (*Oryza sativa* L.) or only vegetative horticultural crops such as onions (*Allium sp*.) and lettuce (*Lactuca sativa* L.) [16]. As the most important horticultural crop globally, we contribute to the expanding body of physiological and phytotoxic knowledge on how microplastics affect tomato plants and their fruit production [23].

## Materials and methods

To investigate the effects of microfibers and their chemical additives, we subjected cherry tomatoes to two microplastic treatments (microfiber and leachate) and a control under greenhouse conditions. Our physical microfiber treatment contained a mixture of four colors of microfibers made from polyester yarn mixed at 0.5% w/w of dry soil. Our chemical leachate treatment involved watering tomatoes with only the chemical extract, or leachate, from the microfibers at a rate that approximated exposure to an equivalent amount of microplastic material as in the physical microfiber treatment. The control cherry tomatoes were grown under the same conditions with no leachate or microfibers added. We grew plants from seed through fruit set for a duration of approximately 3.5 months. We tracked phenology and growth rate through daily observations, regular height measurements, and destructive biomass sampling at key developmental stages.

### Microfiber preparation

To produce PET microfibers, we unraveled polyester yarns (Yarnspirations Caron Kindness 100% polyester yarn, product number 294953 in purple (53006), green (53008), red (53010), and light blue (53004)) into the constituent fibers, then manually cut with micro shears to a target of ≤ 5.0 mm length. The resulting mean length was 3.36 mm and median length was 2.63 mm, although 10% of microfibers ended up longer than targeted (S1 Fig). Although this may have influenced results, this length and fraction of the size distribution is well within limits of what is released in wash water from laundering 100% polyester textiles [24]. Microfiber identity was confirmed to be PET with ATR-FTIR spectroscopy (S3 Fig).

### Soil preparation

We used Pro-Mix BX General Purpose growing medium. This soil contains 79–87% sphagnum moss and 10–14% perlite. We added microfibers at approximately 0.5% soil dry weight concentration (11.4 g of microfibers per 2280 g of dry soil, filling a 12 L pot), keeping microfiber colors at a 1:1:1:1 weight ratio to account for a greater diversity of dyes and potential biological effects.

We microwaved microfibers for three minutes to minimize microbial contamination as in de Souza et al. 2019 [12]. Immediately after, they were incorporated into the soil and mixed for one hour using a cement mixer. We further mixed the soil with a clean drill paint mixer until microfibers were no longer visually distinguishable. A subsample of soil was then examined under a microscope to confirm microfibers were distributed homogeneously. We mixed control soil for an equal time with both the cement mixer and drill paint mixer to account for the effects of mixing on soil structure, and did not microscopically detect any experimental microfibers in subsamples of control soil. All soil mixing was performed in an open-air environment and mixing equipment was cleaned between treatments.

Our concentration (0.5% w/w) is comparable to that of other studies involving plant-microfiber interactions [10–12]. This concentration is higher than reported in a review of microplastics (mainly microfiber) concentrations in the top 10–30 cm of various agricultural soils which noted up to 224.3 mg microplastic/ kg soil (0.02243% w/w) [25]. In a recent review, microfiber counts were found to be underestimated by many extraction methods, with concentrations in the environment likely higher than reported and continuing to increase with added pollution [3]. Therefore, this study is potentially environmentally relevant and likely relevant for future terrestrial systems.

### Leachate preparation and watering regime

To achieve a 0.5% w/w in the microfiber treatment, we distributed 11.4 g of microfibers to each pot. To achieve the same concentration of chemical additive, the leachate concentration was designed such that each pot in the leachate treatment received water from 11.4 g x 1.5 = 17.1 g of fibers in each month through eight watering events. This staggered dosing regime was designed with the assumption that microfibers in soil leach additives slowly, rather than the total concentration being available immediately. The 1.5x factor was chosen to offset potential degradation or incomplete extraction of chemicals that could have resulted in a lower chemical dose than that of the microfiber treatment. We prepared leachate by soaking 6.6 g of microfibers in 1 L of tap water in amber glass bottles for 72 hours at room temperature. This was the largest mass of yarn that could be fully submerged in water. We used amber glass to prevent photodegradation of organic pollutants. Leaching happens most rapidly after initial water contact and declines exponentially thereafter [26,27], so we assumed that 72 hours at this higher dose, with repeated addition to the soil over time, would produce a leachate exposure in line with the physical exposure from microfibers. We then filtered (Melitta 40% bamboo coffee filter) and diluted to produce a final leachate sourced from 1.43 g of microfibers/L. Leachate was poured in a large plastic cooler to be used immediately for that day’s watering. This concentration resulted in each leachate treatment plant receiving the chemicals from 17.1 g microfibers over the course of a month at 1.5 L of watering twice a week (1.43 g/L x 1.5 L x 2 x 4 = 17.1 g of exposure). Plants in the control/microfiber treatments also received 1.5 L of water from the same tap, stored in an identical plastic cooler, at that time. Separate graduated cylinders were used to water the leachate and control/microfiber treatments throughout the experiment.

### Experimental design

We grew the determinate dwarf cherry tomato “Red Robin”. This variety grows well in indoor containers and is photoperiod-insensitive for reproductive cues. We sowed 168 seeds into 160 mL seed starters with 30 g of soil arranged in a Latin square design, with n = 56 seeds for each treatment and the control. Exposure to treatment conditions (microfibers or leachate) began from the experimental onset, with each seed having its own container to negate any effects of neighboring seeds. We checked emergence status (defined as aboveground visibility of shoot) twice a day until new emergence was no longer observed in any cells. We measured shoot height to tip of tallest leaf daily. We rotated trays clockwise daily to minimize microclimate and edge effects. Three weeks after the first seedling had emerged, we randomly chose 84 plants (28 per group) and transplanted into 12 L polyethylene nursery pots with 2.3 kg of soil. We harvested the remaining plants for early-growth biomass measurements. This marked the end of the *seedling* phase (pre-transplant) and beginning of the *vegetative* phase (post-transplant until onset of flowering). We organized the 84 plants into 12 blocks, with 2–3 pots per group in each block. We measured plant height (tip of tallest vegetative structure, excluding any reproductive parts) every 2–3 days. We monitored the emergence of flower buds daily on a per-plant basis, and once flowers developed and opened, the vegetative phase was considered complete. Of the 84 plants, we harvested half once they completed their vegetative phase (n = 14 per group) and half advanced (n = 14 per group) to the *reproductive phase* (flowering until complete fruit set). We split the harvested plants into belowground biomass (roots) and aboveground biomass (photosynthetic or stem material) and dried at 65°C for a minimum of 72 hours. For the remaining plants, we continued height measurements every 2–3 days and assessed fruit ripening qualitatively based on pigmentation. We harvested each plant once all its fruit had ripened, which was approximately 90–100 days after emergence. We separated the plant into fruit, belowground, and aboveground vegetative biomass components for drying at 65°C for a minimum of 72 hours.

### Experimental maintenance

We maintained a 15-hour photoperiod, supplementing natural light when light levels fell below 300 µm/m^2^ with high pressure sodium lights. Ambient conditions were kept at 23 ± 2°C daily, 20 ± 2°C nightly, at 30–60% humidity. In addition to the leachate watering protocol described above, all plants received 1 L of tap water once a week through a two-port manifold irrigation system to maintain consistent soil moisture throughout the experiment. We monitored pests through regular visual inspection, sticky traps, and the use of *Neoseiulus cucumeris* for thrip biocontrol. For maturing plants, we staked with non-treated bamboo stalks and vibrated flowers behind the sepals daily to promote pollination.

### Physical property analysis

We performed water holding capacity analysis by adapting a protocol designed for potting soil [28]. We assessed three control pots against three pots with the same soil containing microfibers used in the experiment. Pot drainage holes were sealed and pots filled with water to measure their total volume. Then, pots were filled with dry soil to the same line. Water was slowly added until saturated; the total water volume added was defined as the total porosity (the combined air and water volume). Then, pots were elevated and pot drainage holes were opened, allowing water to drain. The drained water volume indicated the percent air space. Finally, the water holding capacity was calculated by subtracting the percent porosity from percent air space. We compared bulk density by extracting soil cores (26 cm^3^) from three control pots and three pots containing microfibers. Three vertical and three horizontal cores taken both from the surface soil and 15 cm from the top of the pot. Samples were dried at approximately 50°C for 9 hours in a dehydrator, the time at which mass no longer changed following additional drying.

### Statistical analysis

We executed all graphs and analyses in R version 4.2.1 [29], and ran models with the ‘lme4’ [30] and ‘lmerTest’ [31] packages in R unless otherwise specified. All graphs were produced in ggplot2 [32].

We tracked life cycle progression by monitoring developmental cues during emergence, growth, and fruit set. We tested whether emergence time was significantly different between groups with a One-Way ANOVA. We assessed emergence success with an exact binomial test to determine if seeds met the expectations of the advertised seed mix and Pearson’s Chi-square test to determine if emergence success varied amongst treatments. We assessed flowering time and ripening time with a One-Way ANOVA with blocks as a random effect. To meet assumptions, we checked for normally distributed residuals with a Shapiro test (*P* > 0.05), Q-Q plots, residual plots, as well as equal variance of blocks with Levene’s test (*P* > 0.05). For flowering time, variance was not equal between treatment groups in a Levene’s test (*P* > 0.05). Transformation did not improve the outcome meaningfully. However, F-tests with a balanced design and normal residuals are considered robust against variance heterogeneity, particularly with our low variance ratio [33] of 3.2, so we proceeded with analysis. For ripening time, treatment groups had similar variance and normal distributions with the exception of one extreme outlier. Transformation did not improve the outlier and no similar tests existed to account for block. Therefore, we ran the ANOVA with and without the outlier, which had been growing markedly slower than other plants throughout the experiment and compared the results.

To determine if microfibers affect biomass throughout the lifecycle, we ran linear models for each stage of harvested plants (seedling, vegetative, and reproductive). Models predicted either total dry biomass or shoot to root biomass ratio by age and treatment. For the simpler transplant model, we tested for homoscedasticity with the Breusch-Pagan test (‘lmtest’ package in R [34]), and plotted residuals to check for normality. Assumptions were met so we used an ordinary least squares (OLS) regression. For our vegetative and reproductive data, we ran linear mixed effects models (LMEM) including block as a random effect with a freely varying intercept. LMEM assumptions were checked with residual plots, the variance of random effects, and visualizing homoscedasticity. We also compared models with and without a treatment × age interaction.

For growth rate, we modeled across repeated height measurements of plants from emergence to fruit set. We included only plants present during the whole experiment and used height measurements every five days until the end of the experiment. Since growth appeared asymmetric and sigmoidal, we fit a Gompertz growth curve adapting the process of [35] and using the ‘saemix’ package [36] in R. The curve was defined as: $Y_{it}=K_{i}e^{{-e}^{-A(t-T_{i})}}+\;L_{i}+\;\mu_{it}$, $\mu_{it}\;\sim \;N(\text{0},\sigma^{\text{2}})$ with Y being height over time (t in days since emergence, or age), and estimated parameters K (maximum plant height), L (minimum plant height), A (estimated growth rate) and T (age at inflection point) specified to each individual i.

We assumed all initial growth parameters were 0 (maximum height, growth rate, age at inflection, and minimum height) for the model iterative fitting algorithm, and allowed growth parameters to vary across individuals as covariates. After initial results, we fine-tuned parameters accordingly to maximize curve fit on plot and model fit with Akaike Information Criterion (AIC) [35]. We then performed diagnostics of our Gompertz curve by visually evaluating plots of individual plants and confirming stable, converging estimates of each parameter. We then modified our model to include treatment as a second predictor of maximum height, growth rate, and age at inflection, and compared model fit against the original with AIC.

For both of the soil physical properties, water holding capacity and bulk density, we used Levene’s test to check assumptions followed by a two-sample t-test comparing control soil with soil containing microfibers. We assumed soil physical properties would be unaffected by leachate alone.

## Results and discussion

### Microfibers reduced emergence success

**Fig 1:**
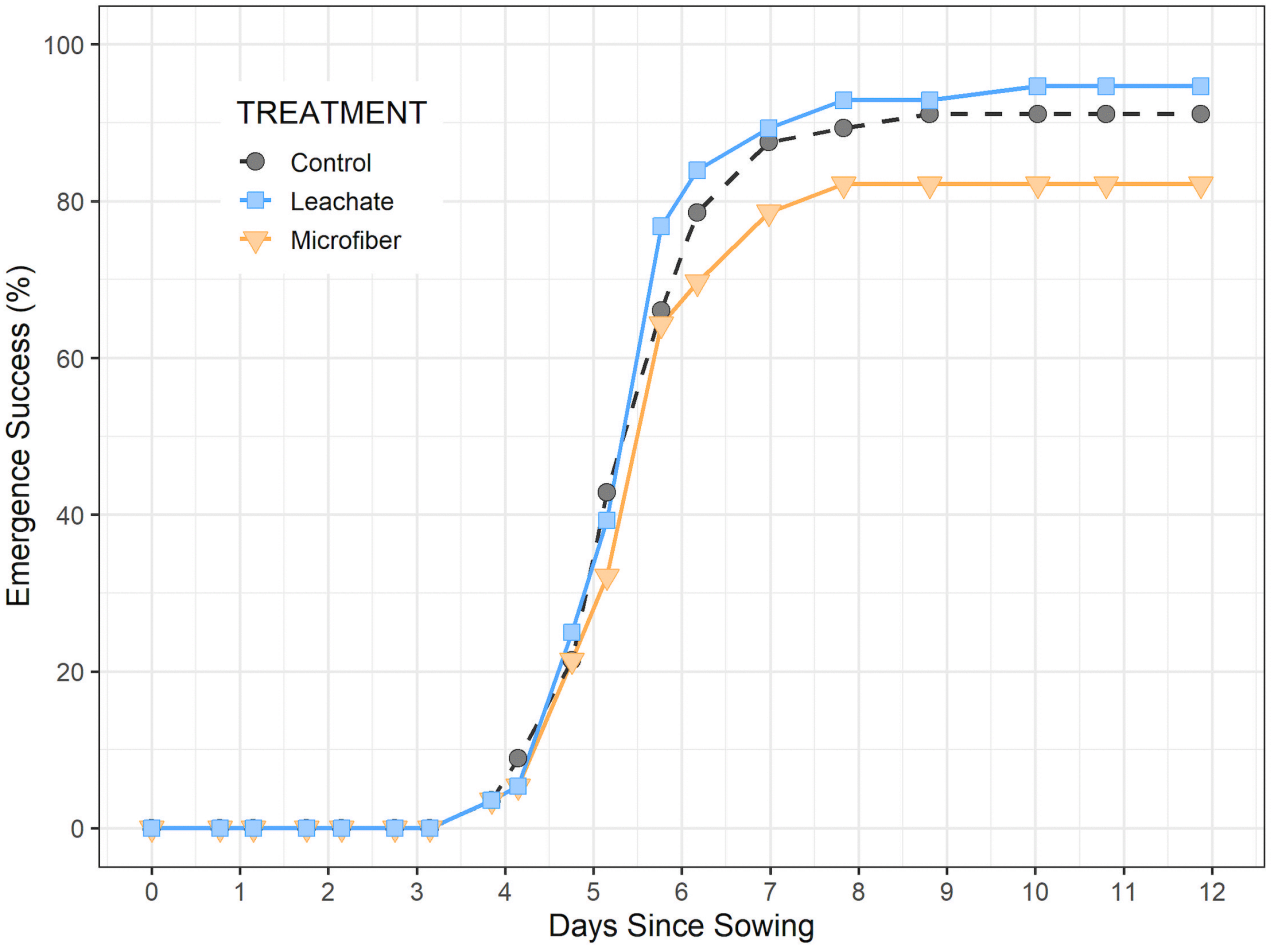
Germination success of cherry tomato seeds. Emergence of plants (n = 56 per group) in soil without microfibers (Control), with microfibers (Microfiber), and with only an aqueous extract from microfibers (Leachate). Plant emergence was monitored daily for 10 days after the last seedling emerged to ensure no late sprouts.

Microfibers negatively impacted emergence success (Fig 1). Emergence success in the microfiber treatment was 11% lower than the expected 93% in a binomial test (82%, n = 56, *P* < 0.01). Emergence success matched expected values for the leachate treatment (95%, n = 56, *P* > 0.05) and the control (91%, n = 56, *P* > 0.05). Despite these varying outcomes, there was no significant difference amongst emergence success of groups in a Pearson’s Chi-squared test (df = 2, χ^2^ = 4.8533, *P* = 0.09). Emergence timing was unaffected by microfibers or leachate in a One-Way ANOVA (*P* > 0.05, n = 56 per group).

While the mechanism is uncertain, negative effects on crop germination have been observed extensively across various kinds and sizes of microplastics [37]. Similar to our results, in a study on perennial ryegrass (*Lolium perenne*), germination was reduced by 7% when seeds were exposed to synthetic fibers (acrylic and nylon composite) relative to the control treatment, although the concentration was considerably lower (0.001% w/w) [38] than in our study (0.5% w/w). In this study, the lack of effect from the leachate treatment suggests a physical interference from the microfibers, such as blocking of seed pore capsules [39] or changes in the soil environment.

### Microfibers altered biomass accumulation over time, but not height

**Fig 2:**
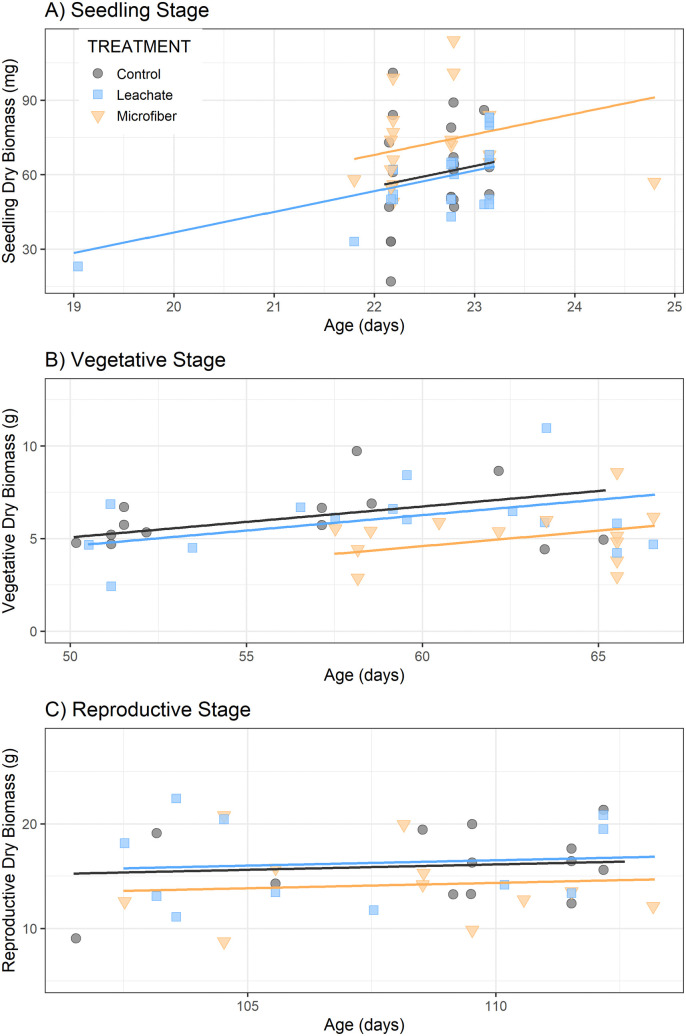
Total dry biomass (g) of cherry tomato, including shoots, roots, and reproductive structures by age. Tomatoes were grown without microfibers (Control), with microfibers (Microfiber), or with an aqueous extract from microfibers (Leachate). Plants were harvested at: a) transplant (n = 23, n = 14, n = 25 respectively due to differing emergence success), b) vegetative stage/onset of flowering (n = 14 per group), or c) reproductive stage/end of life (n = 14 per group). Intercepts are significantly different between Microfiber and other treatments in panels a and b (*P <* 0.05), while panel c only represents trendlines.

Microfibers had variable effects on biomass over time. They positively affected seedling biomass (Fig 2a), with plants producing an average 0.0128 g more biomass than control plants (LMEM, *P* < 0.05) (S1 Table). There was no difference in biomass between the leachate treatment and the control (*P* > 0.05).

In contrast, microfibers negatively affected adult vegetative biomass (Fig 2b), with an average weight 2.14 g lower than control plants (LMEM, *P* < 0.001) (S1 Table). No effect of leachate on biomass was found (LMEM, *P* > 0.05). Upon completion of reproduction, the treatments no longer differed in biomass (P > 0.05) (Fig 2c, S1 Table).

**Fig 3:**
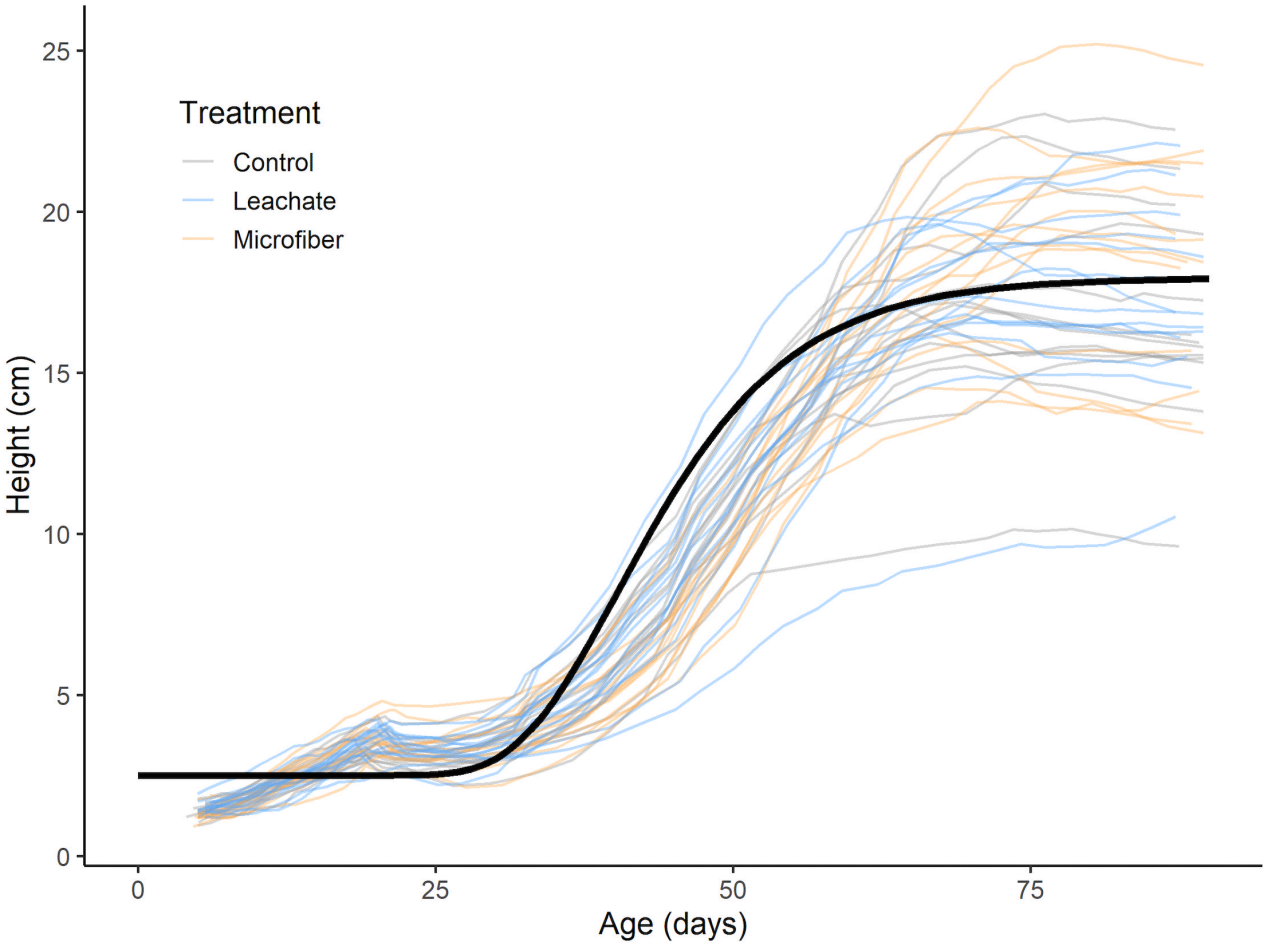
Plant height from emergence to fruit set. Individual plant trajectories are modeled as thinner lines, colored by their treatment. The thicker black line represents a fitted Gompertz curve growth parameters with maximum height *K = *15.45 cm, minimum height *L = *2.51 cm, growth rate *A = *0. 12/day, and maximum growth rate at *T = *40.28 days. Treatment was not a statistically significant predictor of any parameter nor did it improve model fit (pχ^2^ [3] = 0.9749, *P *= 0.81; Δ*AIC *= 6.54; Δ*BIC* = 11.76), suggesting treatment did not influence plant height during the tomato life cycle.

Shoot:root ratio was consistent across treatments (LMEM, *P >* 0.05) in both the vegetative and reproductive stages, suggesting that the reduction in growth was not due to a change in biomass allocation. Furthermore, the difference in biomass did not appear to be driven by growth rate as measured by height (Fig 3). Our best fitting Gompertz model did not include treatment, such that plant height was similar over time across groups and converged to a similar maximum (S2 Table).

Overall, these findings suggest that microfibers in peaty soil do not have lasting effects on plant biomass, height, or resource allocation, but do alter the rate of biomass accumulation. Moreover, the effects of microfibers can vary with plant life stage or growing conditions, as seedlings in small seed starters appeared to benefit from them (Fig 2a) while adults in larger pots suffered (Fig 2b). Due to the lower emergence observed in the microfiber treatment, it is possible that whichever plant properties allowed for successful germination (for example, larger seed size) in the actualized population contributed to a higher average vigor before the plants became more vulnerable to microfiber-related environmental stressors after transplant. Biomass and height effects from microfibers have been similarly variable in the literature. For example, previous research has found no effect on shoot:root ratio or plant height from microfibers at 0.001% w/w in *Lolium perenne* [38]; nor in shoot height of *Silene alba* at 0.1% or 1% w/w [40]. However, in a study on spring onion exposed polyester microfibers at 0.2% w/w, there was a 40% increase in belowground biomass (roots and bulbs) and minimal aboveground effects [12], and a study on carrot found a 27% increase in shoot mass but not root mass exposed to microfibers [10]. Therefore, microfiber effects on biomass appear to be species-specific and/or dependent on dosing, plastic type, and soil conditions.

### Microfibers slowed reproductive development

**Fig 4:**
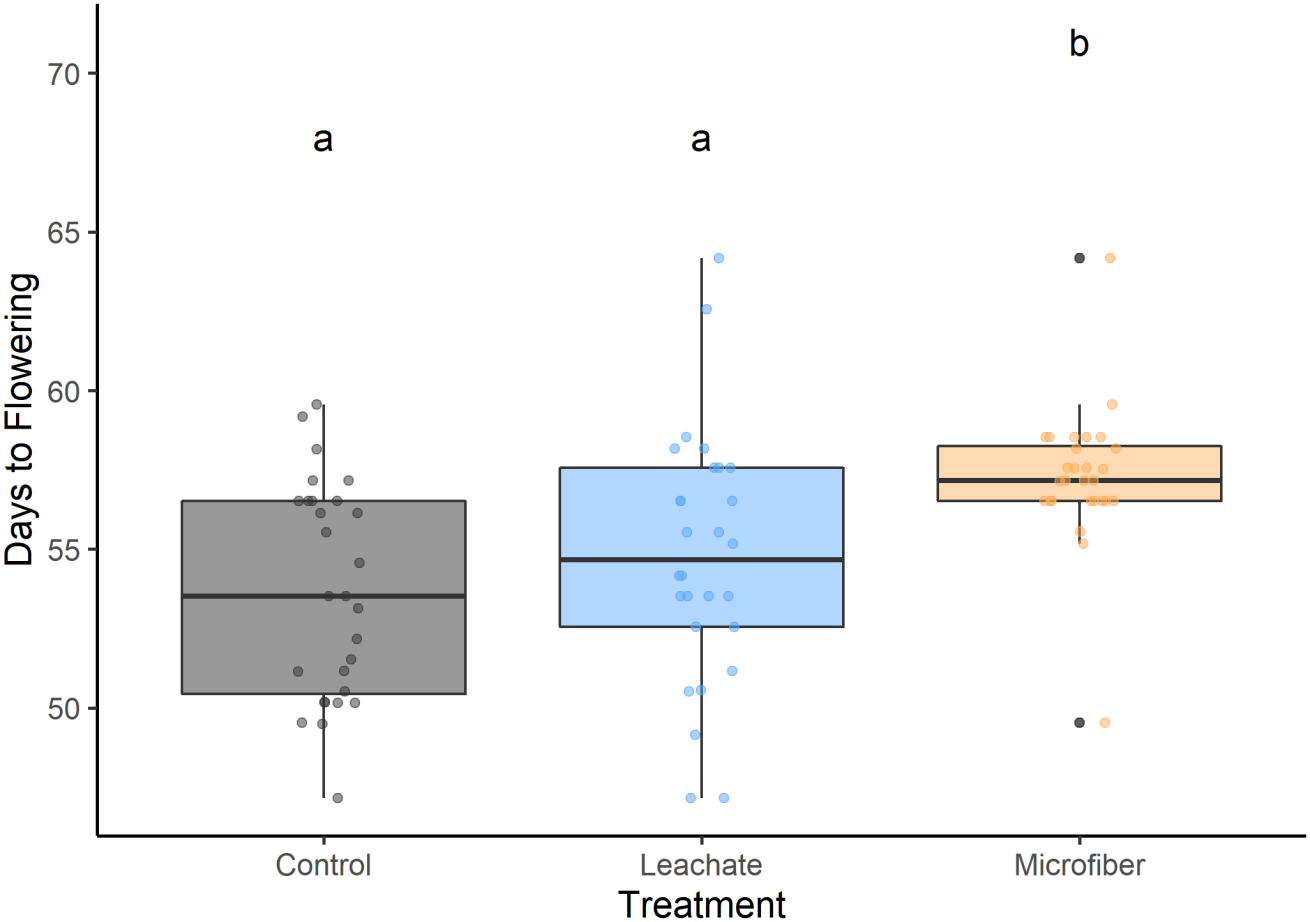
Days to flowering following emergence. Plants (n = 28 per group) in soil without microfibers (Control), with microfibers (Microfiber), and with only an aqueous extract from microfibers (Leachate). Tukey pairwise comparisons of a One-Way ANOVA with blocks are indicated by similar letters (*P* < 0.001 between control and fiber, *P* < 0.05 between fiber and leachate).

Microfibers led to later flowering (Fig 4), with plants in the microfiber treatment flowering an average of 3.6 days later than control plants (One-Way ANOVA, *P* < 0.001) and 2.5 days later than leachate plants (One-Way ANOVA, *P* < 0.05). There was no difference in flowering time between leachate and control plants (One-Way ANOVA, *P* > 0.05).

**Fig 5:**
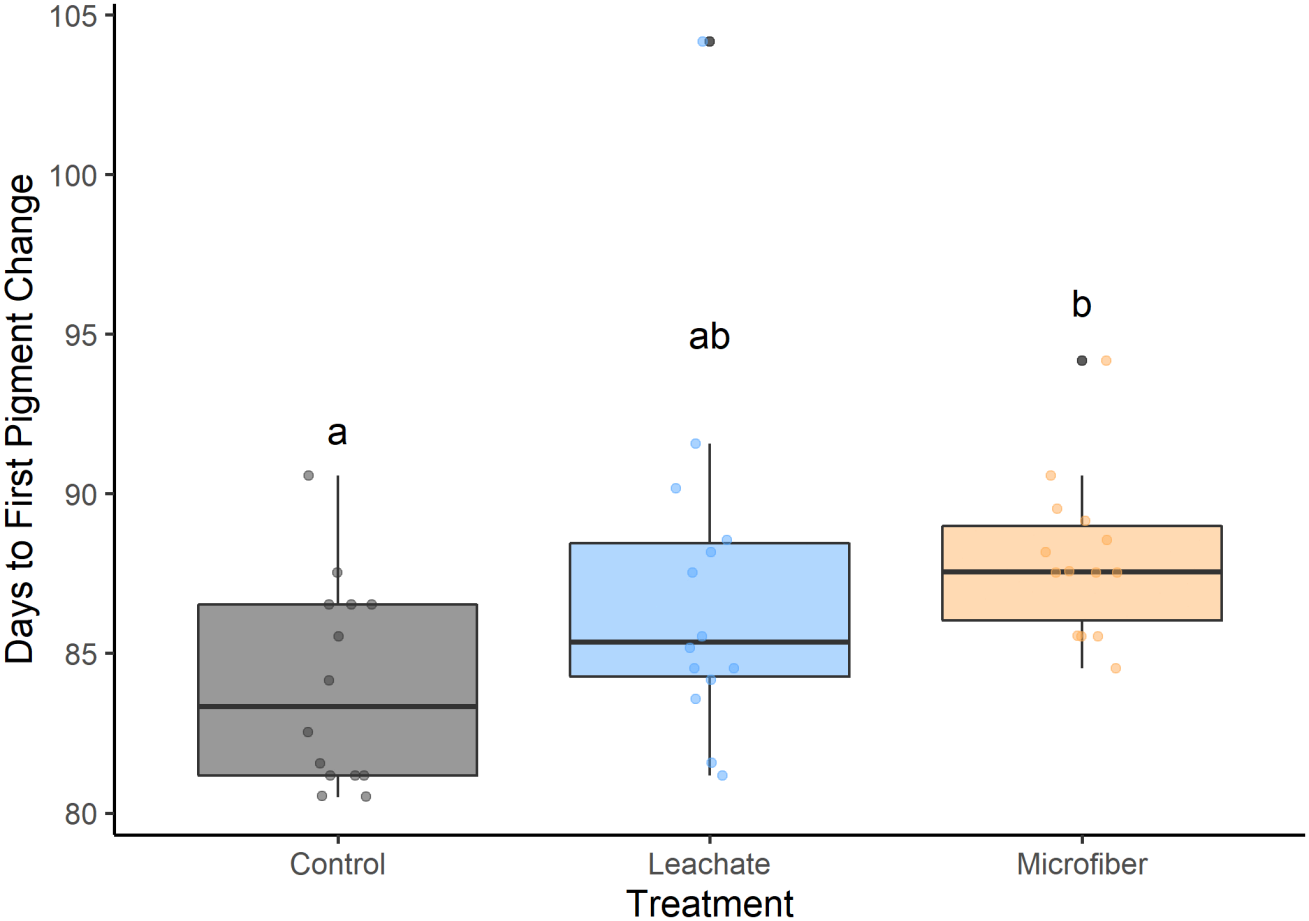
Days to initiation of ripening. Ripening was defined as the first sign of pigment change since emergence (n = 14 per group) in soil without microfibers (Control), with microfibers (Microfiber), and with an aqueous extract from microfibers (Leachate). Tukey pairwise comparisons of a One-Way ANOVA with blocks indicated by similar letters (*P* < 0.05 for significant differences).

Plants exposed to microfibers also had delayed fruit ripening (Fig 5) compared to control plants (+4.0 days, *P* < 0.05).

Since the microfiber plants were also smaller in the vegetative stage (Fig 2b), the phenological delay in flowering and ripening may have been due to needing more time to accumulate the necessary resources. This finding is consistent with a study on larger tomato plants (*Lycopersicon esculentum Mill.*) exposed to sewage sludge containing microplastics. In this study, fewer tomatoes reached maturity in a set time, although it is unknown whether the plants experienced only a delay in fruit production or an absolute reduction in fruit number [23]. In one study on *S. alba* exposed to 0.1% or 1% w/w of microfibers, seed yield was indeed found to be reduced [40].

### Microfibers affected soil properties

**Fig 6:**
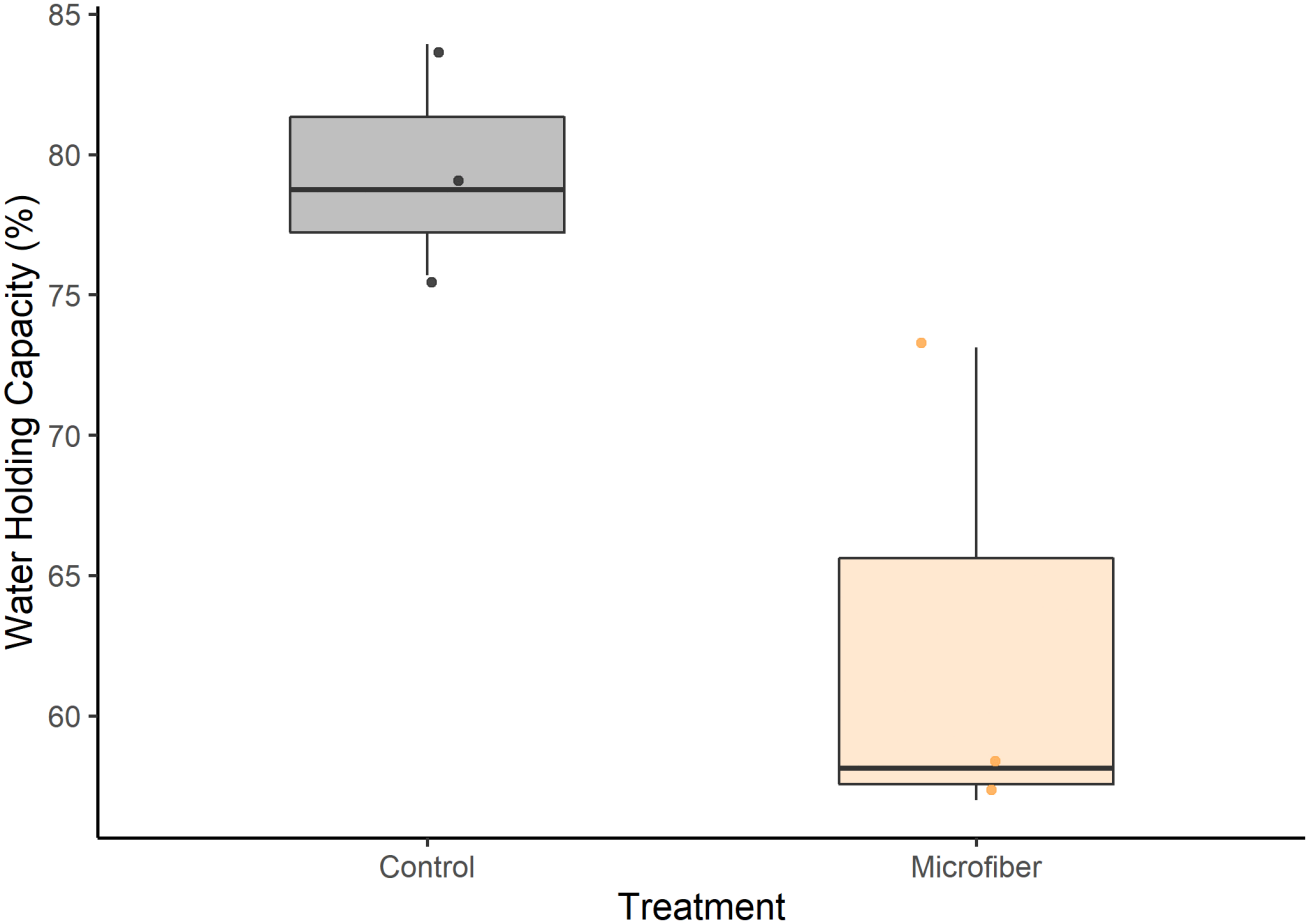
Water holding capacity of potting soil with and without microfibers. A decrease was observed in the water holding capacity of soil with microfibers (Microfiber, n = 3) compared with the control (Control, n = 3)(t-test, (t(4) = 0.2918, *P* < 0.05).

Our findings may be better contextualized by considering soil physical properties in our experiment. We found no difference in soil bulk density between control (0.131 g/cm^3^ ± 0.0024 g/cm^3^) and microfiber (0.124 g/cm^3^ ± 0.011 g/cm^3^) soils; t(4) = 0.865, *P* > 0.05). The lack of effect on soil bulk density was consistent with using clay loam soil in pots at 0.3% w/w [41], but contrast with the decreased soil bulk density observed by de Souza Machado et al. [11] using loamy sand. We attribute our results to the high peat content of our soil overwhelming any potential effects of reduced bulk density on growth, which have been observed in mineral soils. Observed increases in root biomass, as assessed with shoot:root biomass ratios, on plants exposed to microfibers are often attributed to greater root penetration from decreased bulk density [12,42]. The lack of this ratio shift is consistent with the low and similar soil bulk density between treatments. However, we did observe a 16.8% mean decrease in the water holding capacity of soil with microfibers compared with the control (t-test, *P* < 0.05) (Fig 6).

This contrasted with expectations that plastic microfibers would increase water holding capacity by promoting soil aggregation [12,17], although Zhang et al. 2019 [41] also observed this negative effect in a clay loam soil. Hydrophobicity of polyester microfibers may sufficiently increase water repellency of the soil, while also obstructing pores < 30 μm to increase water loss [41]. Moreover, it is possible that root contact with hydrophobic microfibers instead of soil particles may have decreased water absorption by plants, further exacerbating the lower water holding capacity. During biomass processing we noted microfibers were tightly tangled within roots, compared with the more easily removable fibers attached to perlite or peat (S1 Fig). While the proposed mechanisms above were not the focus of this study, they may warrant further research to assess whether lower water availability or even reduced nutrient uptake within microfiber-contaminated soils caused delayed plant growth as discussed in a review of other plant species exposed to microplastics [43]. As these hypotheses rely on the emergent properties of soil and plants, this may also be why negative effects on plants in the microfiber treatment were not observed in the small containers of the seedling stage.

### Environmental implications and future research

This study demonstrates the capacity for polyester microfibers at 0.5% w/w to alter tomato growth by reducing emergence success (Fig 1), slowing biomass accumulation (Fig 2b), and delaying reproductive development (Figs 4,5). Despite the plants growing in favorable soil medium and an indoor environment, these results are overall negative and could have broader implications on crop production, particularly for producers of fruiting dicot plants.

Without knowing the exact chemical composition and concentration of additives in our microfibers, we cannot rule out chemical effects to soils and plants. Nonetheless, the consistent similarity between the leachate treatment and control rejects our hypothesis here that plastic additives from microfibers or their derivatives may mimic hormones such as auxins to influence growth indicators like plant height [20] over this experimental timescale. This suggests that the physical-induced effects of clean microfibers on plant growth observed here may be more important than their chemicals, which has broader implications for interpreting results from other studies using clean, commercial yarn [12,13,17] to investigate effects on soil and plants.

This study demonstrates neutral, positive, and negative effects of microfibers on tomatoes and soil, and reports findings both in line with and contrasting the current literature. It is increasingly understood that this variability is common in terrestrial systems [44], much as it is in aquatic studies [2]. Therefore, we conclude by emphasizing the importance of transitioning into experiments on agricultural fields, that introduce more of the microbial communities, environmental variation, and wastewater and agricultural pollutants that microfibers experience in contact with crops. Recent research has demonstrated that complex synergies can occur when microplastics co-occur with invasive plant species or their legacies, or soil contaminants such as cadmium, amplifying or even altering the direction of effects on plant germination and development [45,46]. Overall, microfiber effects appear to be highly context dependent, and understanding these interactions is of significant environmental and economic importance as microplastics and microfibers continue to accumulate across agricultural soils globally.

## Supporting information

S1 Table Linear model outputs for the seedling stage (Ordinary Least Squares, OLS) and vegetative and reproductive stages (LMEM, Linear Mixed Effects Model) predicting biomass. Estimates are in grams.
(DOCX)

S2 Table Table of fixed effects for Gompertz model predicting growth rate of tomatoes with Age as a predictor and plant identity as a random effect. (DOCX)

S1 Fig Microfiber size distribution. Blue dotted line shows the median and red solid line shows the mean.
(TIF)

S2 Fig Roots entangled with green and red microfibers used in this study, as well as some peat fibers, following extensive rinsing of soil. This tight association between microfibers and roots was commonly observed during biomass assessments.
(TIF)

S3 Fig ATR-FTIR results confirming polyester identity of fibers. (TIF)
