# Supplementary material for: Polyester microfibers delay growth of cherry tomato (Solanum lycopersicum var. cerasiforme) throughout the lifecycle
Source: PLoS One. 2026 Jan 14;21(1):e0336191. doi: 10.1371/journal.pone.0336191 (PMC12803464; doi:10.1371/journal.pone.0336191)
Supplement: S1 Table — Estimates are in grams. (DOCX) [file pone.0336191.s001.docx]

|  | **Seedling**  (OLS, n = 65) | | | **Vegetative**  (LMEM, n = 41) | | | **Reproductive**  (LMEM, n = 35) | | |
| --- | --- | --- | --- | --- | --- | --- | --- | --- | --- |
| **Predictor** | Estimate | CI | P | Estimate | CI | P | Estimate | CI | P |
| Intercept* | -0.127 | -2.69 – 0.151 | 0.079 | -3.28 | -9.00 – 2.44 | 0.252 | 4.82 | -35.1 – 44.7 | 0.807 |
| Treatment (Microfiber) | 0.013 | 0.0215 – 0.234 | **0.019** | -2.14 | -3.32 –  -0.96 | **0.001** | -1.76 | -4.79 – 1.26 | 0.243 |
| Treatment (Leachate) | -0.00186 | -0.117 – 0.0793 | 0.706 | -0.470 | -1.44 – 0.510 | 0.337 | 0.400 | -2.68 – 3.48 | 0.793 |
| Age | 0.00829 | 0.0200 – 0.146 | **0.011** | 0.170 | 0.070 – 0.270 | **0.002** | 0.100 | -0.26 – 0.47 | 0.570 |
| R^2^ | 0.204 |  | | | | | | | |
| R^2^ adjusted | 0.165 |  |  |  |  |  |  |  |  |
| Marginal R^2^ |  | | | 0.234 |  | | 0.071 |  | |
| Conditional R^2^ |  |  |  | 0.613 |  |  | NA† |  |  |
| * The intercept (biomass at Age=0) is not biologically meaningful and is an artifact of the linear model extrapolation.  † The estimated variance for the random effect of block was zero. Therefore, the marginal and conditional R^2^ are identical and only the former is reported. | | | | | | | | | |
