# Supplementary material for: Polyester microfibers delay growth of cherry tomato (Solanum lycopersicum var. cerasiforme) throughout the lifecycle
Source: PLoS One. 2026 Jan 14;21(1):e0336191. doi: 10.1371/journal.pone.0336191 (PMC12803464; doi:10.1371/journal.pone.0336191)
Supplement: S2 Table — (DOCX) [file pone.0336191.s002.docx]

| **Fixed effects** | | | |
| --- | --- | --- | --- |
| **Parameter** | **Estimate** | **SE** | **CV (%)** |
| Total Growth | 15.45 | 0.512 | 3.3 |
| Approach | 0.12 | 0.006 | 5.2 |
| Timing | 40.28 | 0.554 | 1.4 |
| Lower Asymptote | 2.51 | 0.042 | 3.3 |
| **Likelihood computed by importance sampling** | | | |
| -2LL = 957.811 | | | |
| AIC = 979.574 | | | |
| BIC = 998.6883 | | | |
